# Whole body vibration exercise combined with an extract of *Coriandrum sativum* modify some biochemical/physiological parameters in rats

**DOI:** 10.1042/BSR20170070

**Published:** 2017-06-07

**Authors:** Éric H.F.F. Frederico, André L.B.D. Cardoso, Carlos A.S. Guimarães, Lívia P. Almeida, Rosane F. Neves, Danúbia C. Sá-Caputo, Eloá Moreira-Marconi, Carla F. Dionello, Danielle S. Morel, Laisa L. Paineiras-Domingos, Rebeca G. Costa-Cavalcanti, Cintia R. Sousa-Gonçalves, Adriano Arnóbio, Nasser R. Asad, Mario Bernardo-Filho

**Affiliations:** 1 Programa de Pós-graduação em Biociências, Instituto de Biologia Roberto Alcantara Gomes, Universidade do Estado do Rio de Janeiro, Rio de Janeiro, RJ 20551-030, Brasil; 2 Programa de Pós-Graduação em Ciências Médicas, Faculdade de Ciências Médicas, Universidade do Estado do Rio de Janeiro, Rio de Janeiro, RJ 20551-030, Brasil; 3 Laboratório de Vibrações Mecânicas e Práticas Integrativas e Complementares, Departamento de Biofísica e Biometria, Instituto de Biologia Roberto Alcantara Gomes, Universidade do Estado do Rio de Janeiro, Rio de Janeiro, RJ 20551-030, Brasil; 4 Programa de Pós-Graduação em Fisiopatologia Clínica e Experimental, Faculdade de Ciências Médicas, Universidade do Estado do Rio de Janeiro, Rio de Janeiro, RJ 20551-030, Brasil

**Keywords:** biodistribution, biomarkers, body mass, coriander, feed intake, Whole body vibration

## Abstract

The aim of the present study was to evaluate the effect of the association of whole body vibration (WBV) exercise with an aqueous extract of coriander on the biodistribution of the radiopharmaceutical sodium pertechnetate, on the concentration of some plasma biomarker, on the feed intake, on the body mass, and on the stool consistency in rats. Rats were divided in four groups and submitted to different treatments for 40 days. The control group (CON) received deionized water. The group treated with coriander (COR) received the extract of coriander. The rats that were exposed to WBV exercises (WBV-E) also received deionized water. A group of animals received coriander and was exposed to WBV (COR + WBV-E). We found in testis a decrease (0.13 ± 0.01 to 0.06 ± 0.03) of the percentages of injected radioactivity per gram (%ATI/g) in the WBV-E in comparison with the COR. There is no significant alteration on the concentrations of the plasma biomarkers. The feed intake showed a statistically significant increase in WBV-E. No significant difference on the body mass was found. The stool analysis showed a statistical difference on the consistency between COR (hard and dry, darker) and all the other groups (normal). In conclusion, it was verified that possible modifications in some biochemical/physiological parameters of the rats submitted to WBV exercise would be capable to increase the feed intake without changing the body mass, and normalizing the stool consistency altered by the coriander supplementation. Further studies are needed to try to understand better the biological effects involving the association of WBV exercise and coriander.

## Introduction

Mechanical vibrations can be defined as a physical agent with harmonic oscillatory motion about an equilibrium point. They can be artificially produced in oscillating/vibratory platforms [1–3]. Furthermore, they can be transmitted to the body when there is a direct contact of the subject with the base of this platform in operation. The exposure to these vibrations generated in oscillating/vibratory platform produces whole body vibration (WBV) exercises [1,4].

The clinical use of the WBV exercises has been reviewed and important findings described [1,3] such as increased muscle strength and power, improved balance, increased bone mineral density [5–7], aspects related to quality of life and decrease the risk of falls [8,9]. In addition, a non-health promoting effects on blood profile was reported by Theodorou et al. [10]. However, undesirable effects have been reported in a revision (Sá-Caputo et al., 2015) as hot feet, itching of the lower limbs, vertigo [11], severe hip discomfort, pain of jaw, neck and lower limbs [12], and hematuria [13].

Rauch et al. [2] have discussed several parameters related to the use of WBV exercises and that must be considered. In consequence, the biomechanical parameters such as frequency, amplitude, and peak acceleration must be selected and adapted to the characteristics of each individual. In controlled conditions, the vibrations generate WBV exercise in good and safe conditions [3]. In addition, it should be set as a working time interspersed with a rest time [2–4].

The effect of the WBV exercises on the concentrations of some biomarkers has been investigated in human beings [14,15] and in animals [16–18].

As the use of the WBV exercises has been increased (see PubMed database, using the keyword ‘whole body vibration’), the development of experimental models to evaluate, in a controlled manner, the effect of these exercises in organs and tissues is desirable. One of the possible ways to study these vibrations in tissues/organs, it is by evaluating the biodistribution of radiopharmaceuticals, as already described for physical activity [19], chemical [20], and physical (laser) agents [21]. Pereira et al. [22] (vibration with 20 Hz) and Frederico et al. [16] (vibration with 12 Hz) reported the effect of WBV exercise in the biodistribution of radiopharmaceuticals in rats using a side-alternating platform.

Authors have investigated the effect of the WBV exercise in association with some substances [23–26], including natural products [16,17].

Natural products have been used by human beings as food sources and as medications [27]. However, the mechanism of action and the efficacy of these natural products in most cases must be validated scientifically [28]. *Coriandrum sativum* (coriander) is an herbaceous plant that has been used in the management of patients with diabetes [29,30]. It is originally from the Eastern Mediterranean region, belonging to family *Apiaceae* [31]. Furthermore, it is grown in a wide range of environmental conditions [32]. It is cultivated for its aromatic leaves and seeds in North Africa, Central Europe, and Asia as a spice and a medicine [33]. Coriander is known to possess antifungal [34], antibacterial [35], and antioxidant [36] properties. In traditional medicine, it is used for gastrointestinal complications such as dyspepsia, flatulence, diarrhea, vomiting [37], and as an antiseptic and emmenagogue [38].

The aim of the present study was to evaluate the effect of the association of WBV exercise with an aqueous extract of coriander on the biodistribution of the radiopharmaceutical sodium pertechnetate (Na^99m^TcO_4_), on the concentration of some plasma biomarkers, on the body mass, on the feed intake, and on the stool consistency in *Wistar* rats.

## Material and methods

### Animals and ethical approach

Adult Wistar rats (*n*=20, 245–280 g) aging from 3 to 4 months. The animals were kept under care at average temperature of 25°C, relative humidity approximately 55% and light/dark cycle of 12 h and were fed with standard diet and water *ad libitum*. All experiments were conducted following the standards of the *Comitê de Ética Para o Uso de Animais Exprimentais* (CEUA), *Instituto de Biologia Roberto Alcantara Gomes* (IBRAG) that has approved the investigation (CEUA/041/2013).

### Preparation of the extract of coriander

A commercial dry seed of coriander (*C. sativum*) was used (lot 075, validity up to May 2017, *Distribuidora de Cereais Crowne Ltda*, Rio de Janeiro). This natural product was chosen because it is also used as a medicinal plant. To prepare the extract, 80 mg of *C. sativum* was added to 10 ml of deionized water. Then, the preparation was vortexed for 1 min, centrifuged (clinical centrifuge, 15000 rpm, 15 min), and the supernatant was considered to be at a concentration of 8 mg/ml. The quality control of the preparation of the extract was controlled with spectrophotometer analysis (extract with optical density at 480 nm), as previously published [39].

### Experimental procedures

The WBV exercise was performed everyday between 7.00 and 9.00 a.m. on a synchronous platform (Globus G-Vibe 800, Italy), which generated sinusoidal vertical vibrations at 50 Hz frequency, 0.78 mm amplitude, and peak acceleration of 7.84 g.

The Wistar rats (*n*=20) were divided equally in four groups. The control group (CON), that received by gavage [40] 1.0 ml of deionized water. The coriander group (COR), where animals received 1.0 ml of aqueous extract (8 mg/ml) also by gavage. The rats that were submitted to the vibration generated in the platform (WBV-E) also received 1.0 ml of deionized water. Animals of the group (COR + WBV-E) received 1.0 ml of coriander (8g/ml) and were submitted to vibration generated in the platform. The animals received saline (group CON) or coriander extract (groups COR and COR + WBV-E) daily during 40 consecutive days. The animals of the groups WBV-E and COR + WBV-E were submitted every day during 40 consecutive days to vibrations generated in the platform.

**Figure 1:**
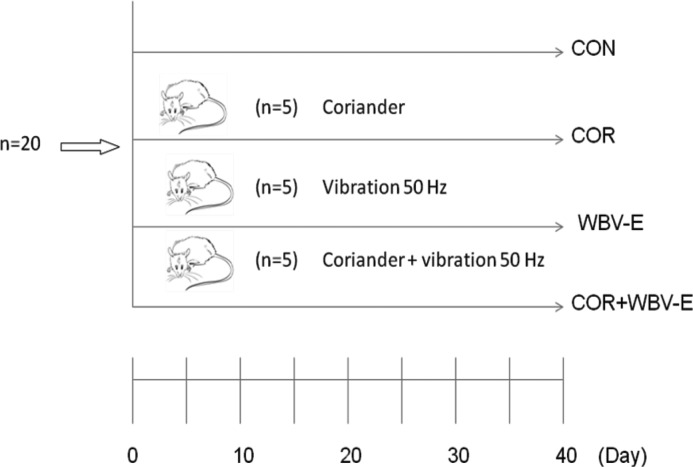
Experimental design. 20 Wistar rats were divided randomly in four groups, each one, in a cage. 1) CON - group received by gavage deionized water. 2) COR - received by gavage coriander. 3) WBV-E - received by gavage deionized water and were submitted to vibration generated in platform, 50Hz, 0.78 mm and 7.84 g peak acceleration or 4) COR+WBV-E - received bay gavage coriander and were submitted to vibration generated in platform, 50Hz, 0.78 mm and 7.84 g peak acceleration

The scheme of distribution of the groups is depicted in the experimental design that is shown in Figure 1.

**Figure 2:**
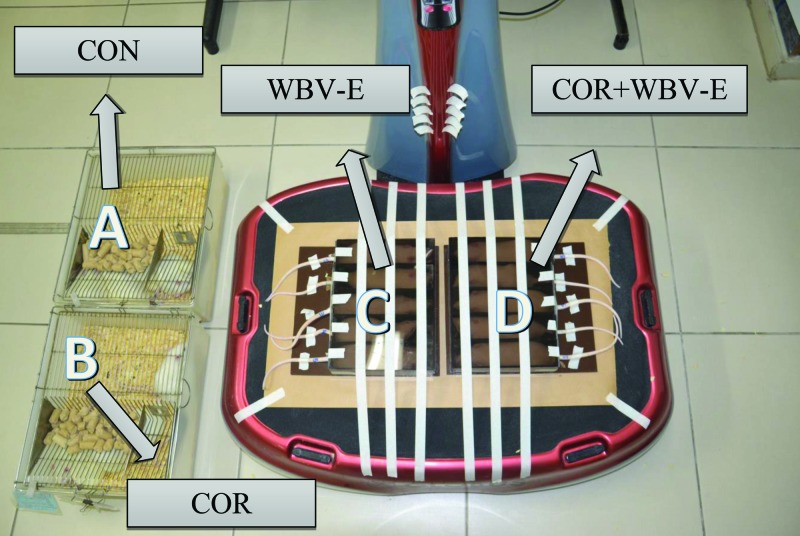
*Wistar* rats on (C and D) and close (A and B) of the platform CON - control group; COR - group treated with coriander; WBV-E - whole body vibration exercise group; and COR+WBV-E - group treated with coriander and submitted to whole body vibration exercise.

The work time of the animals in the platform was 5 min, considering four bouts of 30 s separated by 1-min rest intervals. The animals were put in a man-made acrylic base fixed in the teeterboard of the platform with tape, as it is shown in Figure 2. Every day the animals of CON and COR groups were put close to the platform (approximately 30 cm) that was turn on, to avoid a possible bias of stress provoked by sound and other factors of the surrounding of the synchronous platform. However, the animals did not have a direct contact with the platform (Figure 2). This investigation used a similar frequency and total time per day reported by Pawlak et al. [18] in the treatment of the rats on the platform.

### Administration of the radiopharmaceuticals and blood samples collection for biochemical analysis

One day after the different treatments (41th day), the animals were anesthetized with sodium thiopental. Just after the animals’ anesthesia effect, the radiopharmaceutical Na^99m^TcO_4_ (1.85 MBq/ml) was administrated 0.3 ml (550 kBq) via ocular plexus. After 10 min, sample of blood was obtained from by cardiac puncture and used for biochemical analysis. Following, the animals were killed (CO_2_ asphyxiation) (CONCEA, 2013), the organs (thyroid, stomach, bowel, kidney, liver, pancreas, brain, bone, lung, heart, spleen, muscle, penis, prostate, seminal vesicle, bladder, testis, and blood) were isolated, the radioactivity determined in a well counter, and the percentages of injected radioactivity per gram (%ATI/g) in the organs were calculated as reported elsewhere [22].

Sample of whole blood (without anticoagulant) was also withdrawn to determine the concentrations of selected plasma biomarkers (glucose, urea, creatinine, cholesterol, triglyceride, high-density lipoprotein (HDL), aspartate aminotransferase (AST), alanine aminotransferase (ALT), alkaline phosphatase, total bilirubin, direct bilirubin, amylase, lipase, creatine kinase (CK), calcium, magnesium, total protein, and albumin), were then measured in a clinical laboratory of the *Universidade do Estado do Rio de Janeiro*. The determinations were performed in automated equipment (COBAS INTEGRA 400 plus, Roche, Basel, Switzerland).

### Body mass analysis

All the animals were weighed weekly on a digital balance (FILIZOLA BP6, São Paulo, Brazil). The mass of each animal was determined. The mass of the animals of each group was determined by percentage (%). The % was calculated as the ratio between the mass of animals in each week with the first day, and multiplied by 100.

### Feed intake analysis

The feed intake was measured daily in each group. Five hundred grams of feed was offered daily. In the next day, the left feed was determined on a digital balance (FILIZOLA BP6, São Paulo, Brazil). The feed intake was calculated by the difference between 500 g and the left feed in each day. Following, the quantity of feed was completed daily to 500 g.

### Stool consistency analysis

**Figure 3:**
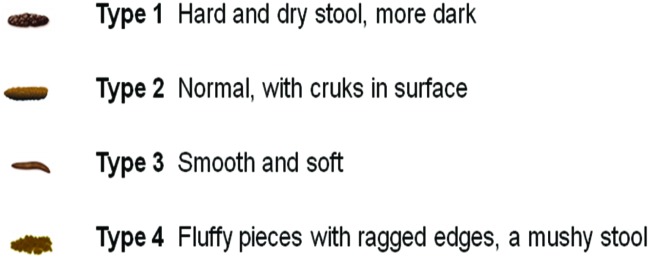
Bristol stool form scale adapted for *Wistar* rats

A stool consistency was made using a Bristol stool form scale [41] adapted as depicted in Figure 3. Three different, independent, and blinded evaluators have analyzed daily and qualitatively the consistency of the stool and according to the scale, they choose a value (Figure 3). The median of these three analyses was considered. The stool sample was collected before the gavage of the animals.

### Statistical analysis

A normality test was done to determine if the data set is well-modeled by a normal distribution. As all the studied data do not follow a normal distribution, a Kruskal–Wallis test following the post-test Student–Newman–Keuls was done, for the statistical analysis of the results with BioEstat 5.3 (Instituto Mamiraua, Pará, Brasil). Data are presented as mean ± standard deviation (±SD), median ± interquartile range (IQR), or as percentage (%). Statistical significance was accepted at *P*<0.05. Epsilon-squared (*ɛ*^2^) were analyzed to estimate the effect size. The *ɛ*^2^ assumes the value from 0 (indicating no relationship) to 1 (indicating a perfect relationship). The values approximately 0.5 were considered in the present study as a moderate relationship. Effect sizes were analyzed to determine the magnitude of an effect independent of sample size as reported elsewhere (Tomczak and Tomczak, 2014). The *ɛ*^2^ effect sizes were measured by the following formula:

$$\varepsilon^{2}=\frac{H}{n^{2}+1/n+1}$$

Where *H* is the value obtained in the Kruskal–Wallis test (the Kruskal–Wallis *H*-test statistic) and *n* is the total number of observations.

## Results

Table 1 shows the %ATI/g of the Na^99m^TcO_4_ in the various organs isolated from the animals submitted to different treatments. It is possible to verify that in testis there was a significant (*P*=0.0032) alteration in the uptake of radiopharmaceutical in the animals submitted to the vibration (WBV-E group) in comparison with the COR group, with moderated relationship (*ɛ*^2^ = 0.5581). In the other organs, there is no alteration statistically significant and the *ɛ*^2^ is small.

**Table 1:** %ATI/g of the Na^99m^TcO_4_ in the various organs isolated from the animals submitted to different treatments

Organs	CON	COR	WBV-E	COR + WBV-E	*P*	*ɛ*^2^
Thyroid	3.87 ± 1.98	3.14 ± 1.05	2.28 ± 1.30	3.34 ± 1.06	0.5758	0.1240
Stomach	1.36 ± 0.22	2.15 ± 1.26	1.46 ± 0.81	1.64 ± 0.91	0.4267	0.1545
Bowel	0.29 ± 0.10	0.35 ± 0.18	0.20 ± 0.10	0.31 ± 0.12	0.4885	0.1619
Kidney	0.34 ± 0.07	0.37 ± 0.12	0.29 ± 0.06	0.34 ± 0.06	0.5857	0.1210
Liver	0.38 ± 0.06	0.49 ± 0.14	0.42 ± 0.09	0.53 ± 0.17	0.2374	0.2352
Pancreas	0.12 ± 0.04	0.18 ± 0.03	0.14 ± 0.04	0.14 ± 0.05	0.3122	0.2548
Brain	0.01 ± 0.00	0.01 ± 0.00	0.01 ± 0.00	0.02 ± 0.01	0.6474	0.1271
Bone	0.08 ± 0.03	0.11 ± 0.05	0.07 ± 0.02	0.09 ± 0.03	0.3221	0.1939
Lung	0.40 ± 0.12	0.47 ± 0.15	0.32 ± 0.05	0.42 ± 0.09	0.2658	0.2200
Heart	0.19 ± 0.07	0.19 ± 0.07	0.16 ± 0.05	0.17 ± 0.05	0.8711	0.0394
Spleen	0.22 ± 0.05	0.20 ± 0.09	0.16 ± 0.04	0.21 ± 0.06	0.4794	0.1376
Muscle	0.07 ± 0.01	0.08 ± 0.02	0.05 ± 0.01	0.06 ± 0.02	0.3943	0.2486
Penis	0.41 ± 0.04	0.40 ± 0.05	0.32 ± 0.06	0.41 ± 0.06	0.2491	0.2941
Prostate	0.17 ± 0.02	0.22 ± 0.07	0.15 ± 0.04	0.19 ± 0.02	0.0921	0.4952
Seminal vesicle	0.14 ± 0.04	0.11 ± 0.03	0.11 ± 0.05	0.11 ± 0.01	0.4840	0.1362
Bladder	0.31 ± 0.09	0.26 ± 0.10	0.27 ± 0.10	0.25 ± 0.08	0.9177	0.0421
Testis	0.09 ± 0.01	0.13 ± 0.01^*^	0.06 ± 0.03	0.10 ± 0.03	0.0302	0.5581
Blood	1.00 ± 0.27	1.13 ± 0.49	0.93 ± 0.23	1.24 ± 0.14	0.5133	0.1351

COR + WBV-E, group treated with coriander and submitted to vibration; WBV-E, group submitted to vibration generated in platform. Values are shown as the means ± SD. Adjusted *P* values (Student–Newman–Keuls correction) were considered statistically significant at *P*<0.05. *P<0.01 compared with WBV-E; *ɛ*^2^, epsilon squared.

After 40 days of treatment, there is no alteration (*P*>0.05) on concentrations of some plasma biomarkers (Table 2) in comparison with the CON, independently on the type of treatment (only Coriander or WBV, or with the association coriander and WBV). The *ɛ*^2^revealed values from 0.0204 to 0.3279, indicating a small relationship.

**Table 2:** Concentration of some plasma biomarkers that was determined in the animals submitted to different treatments

	CON	COR	WBV-E	COR+WBV-E	*P*	*ɛ*^2^
Glucose (mmol/l)	7.04 ± 0.90	6.78 ± 0.78	6.35 ± 0.56	6.33 ± 0.30	0.4211	0.1759
Urea (mmol/l)	7.93 ± 0.88	7.73 ± 0.51	7.97 ± 0.88	8.53 ± 1.14	0.6050	0.1026
Creatinine (µmol/l)	39.78 ± 4.42	35.36 ± 6.18	38.89 ± 4.42	35.36 ± 0.08	0.3391	0.1868
Cholesterol (mmol/l)	1.20 ± 0.23	1.26 ± 0.05	1.13 ± 0.19	1.13 ± 0.08	0.3186	0.2512
Triglyceride (mmol/l)	0.53 ± 0.08	0.51 ± 0.13	0.43 ± 0.08	0.41 ± 0.01	0.2371	0.3259
HDL (mmol/l)	1.06 ± 0.13	1.14 ± 0.03	1.23 ± 0.09	1.13 ± 0.15	0.1778	0.3279
AST (µKat/l)	2.14 ± 0.36	2.01 ± 0.45	2.06 ± 0.70	2.21 ± 0.80	0.9442	0.0224
ALT (µKat/l)	1.40 ± 0.24	1.19 ± 0.08	1.19 ± 0.12	1.15 ± 0.15	0.4565	0.1533
Alkaline phosphatase (µKat/l)	2.15 ± 0.45	1.90 ± 0.37	1.81 ± 0.42	1.73 ± 0.36	0.8199	0.0543
Total bilirubin (µmol/l)	1.37 ± 0.34	1.20 ± 0.34	1.37 ± 0.51	1.54 ± 0.34	0.8389	0.0469
Direct bilirubin (µmol/l)	0.68 ± 0.34	0.51 ± 0.17	0.86 ± 0.34	0.86 ± 0.17	0.3349	0.2424
Amylase (µKat/l)	44.08 ± 2.05	48.05 ± 8.06	44.91 ± 6.52	45.18 ± 5.58	0.9471	0.0204
Lipase (µKat/l)	0.11 ± 0.01	0.10 ± 0.01	0.10 ± 0.01	0.10 ± 0.01	0.5368	0.1209
CK (µKat/l)	25.19 ± 6.39	24.15 ± 5.91	25.46 ± 5.74	18.48 ± 3.59	0.3047	0.2790
Calcium (mmol/l)	2.40 ± 0.15	2.35 ± 0.07	2.38 ± 0.10	2.40 ± 0.10	0.8122	0.0530
Magnesium (mmol/l)	1.07 ± 0.16	0.95 ± 0.08	0.95 ± 0.08	0.95 ± 0.04	0.5569	0.1153
Total protein(g/l)	57.0 ± 2.0	57.0 ± 3.0	57.0 ± 3.0	60.0 ± 3.0	0.5147	0.1272
Albumin (g/l)	34.0 ± 2.0	33.0 ± 2.0	33.0 ± 1.0	36.0 ± 3.0	0.3822	0.1801

COR + WBV-E, group treated with coriander and submitted to vibration; WBV-E, group submitted to vibration generated in platform. Values are shown as the means ± SD; *ɛ*^2^, epsilon squared.

**Figure 4:**
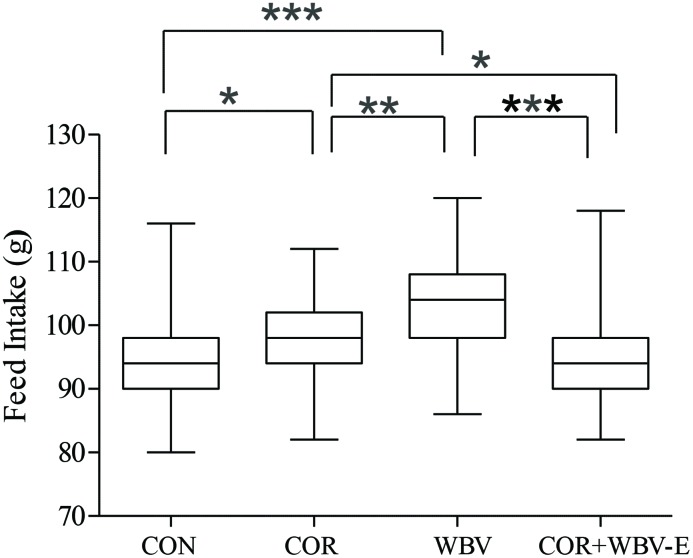
Feed intake (g) of animals submitted to different treatments. Adjusted *p* values (Student-Newman-Keuls correction) were considred statistically significant at *p < 0.05, **p < 0.01, and ***p < 0.001.

Considering the effect of 40-day WBV and its association with coriander on feed intake in rats treated with coriander, there is a significant difference on feed intake (Figure 4). The animals of the WBV-E group have more feed intake than that of the others groups. The CON and COR + WBV-E have the lowest feed intake. The *ɛ*^2^ (not shown in the figure) was 0.2134, indicating small relationship.

The Table 3 shows the body mass (%) of the animals submitted to different treatments. The results showed no alterations among the groups in comparison with the CON, independently on the type of treatment (only coriander or WBV, or with the association coriander and WBV). The values of *ɛ*^2^ varied from 0.3081 to 0.3893, indicating small relationship.

**Table 3:** The body mass (%) of the groups of animals submitted to different treatments

Week(s)	CON	COR	WBV-E	COR + WBV-E	*P*	*ɛ*^2^
0	100.00 ± 0.00	100.00 ± 0.00	100.00 ± 0.00	100.00 ± 0.00	0.1003	0.3286
1	100.15 ± 9.44	103.78 ± 7.11	103.29 ± 5.19	102.58 ± 5.50	0.0661	0.3784
2	102.22 ± 9.25	105.82 ± 7.30	106.43 ± 6.74	104.52 ± 6.48	0.0603	0.3893
3	103.10 ± 8.49	106.99 ± 8.06	109.86 ± 7.70	107.58 ± 7.27	0.0899	0.3418
4	105.02 ± 10.1	109.32 ± 8.75	111.57 ± 7.33	109.35 ± 8.22	0.1190	0.3081
5	106.79 ± 9.79	110.19 ± 10.5	114.29 ± 7.47	110.48 ± 7.55	0.1180	0.3090

COR + WBV-E, group treated with coriander and submitted to vibration; WBV-E, group submitted to vibration generated in platform. Values are shown as %; *ɛ*^2^, epsilon squared.

Considering the effect of 40-day WBV and its association with coriander on stool consistency, the results showed statistical difference (*P*<0.05) on the stool consistency between COR and all the other groups (CON, WBV-E, and COR + WBV-E) as shown in Table 4. The *ɛ*^2^ was 0.6571 indicating a moderated relationship.

**Table 4:** Stool consistency of animals submitted to different treatments

Day(s)	CON	COR	WBV-E	COR + WBV-E	*P*	*ɛ*^2^
1–10	2.00 ± 0.00*	1.00 ± 1.00	2.00 ± 0.00^†^	2.00 ± 0.00*	0.0024	0.3697
11–20	2.00 ± 0.00^‡^	1.00 ± 0.00	2.00 ± 0.00^‡^	2.00 ± 0.00^†^	<0.0001	0.8154
21–30	2.00 ± 0.00^‡^	1.00 ± 0.00	2.00 ± 0.00^‡^	2.00 ± 0.00^‡^	<0.0001	0.8946
31–40	2.00 ± 0.00^‡^	1.00 ± 0.00	2.00 ± 0.75^‡^	2.00 ± 0.00^‡^	<0.0001	0.8222
Total (1–40)	2.00 ± 0.00^‡^	1.00 ± 0.00	2.00 ± 0.00^‡^	2.00 ± 0.00^‡^	<0.0001	0.6571

COR + WBV-E, group treated with coriander and submitted to vibration; WBV-E, group submitted to vibration generated in platform. Values are shown as the median ± IQR. Adjusted *P* values (Student–Newman–Keuls correction) were considered statistically significant at *P<0.05, ^†^*P*<0.01, and ^‡^*P*<0.001. * compared with COR group; *ɛ*^2^, epsilon squared.

## Discussion

Experimental models involving the evaluation of effects of WBV exercise in animals that consumed some substances [17,24,26] are very relevant and have stimulated our investigation. New models regarding the effect of other synthetic and natural products in association with WBV exercise using different biomechanical parameters and time of exposition are desirable. Naghii et al. [17] have evaluated the effect of consumption of some natural medicinal products (fatty acids, vitamin D, and boron) with WBV (10–50 Hz). In the present study, we hypothesized that the combination of WBV exercise and coriander supplementation could potentialize their biological effects in rats. In consequence, the effects of the treatments with WBV exercise and a medicinal product (coriander) extract on the biodistribution of the radiopharmaceutical Na^99m^TcO_4_, on the concentration of some plasma biomarkers, on the feed intake, on the body mass, and on the stool consistency in Wistar rats were assessed.

The pattern of the biodistribution of a radiopharmaceutical, in general, depends on physiological characteristics of an organ/tissue [42]. Previously, Pereira et al. [22] have shown that, in rats, the exposure to vibration with 20 Hz can alter the uptake of a radiopharmaceutical in some organs. Frederico et al. [16] reported that the association between coriander and WBV (12 Hz) increases the uptake of Na^99m^TcO_4_ in spleen. The determination of the uptake of the Na^99m^TcO_4_ in different organs (Table 1) permitted to verify the effect of the different treatments in some organs. The effect in the testis (Table 1) is only in animals of the COR group compared with rats of the WBV-E group, with an increase in the uptake of the radiopharmaceutical. In 2010, Sharma et al. [43] have reported the prophylactic efficacy of coriander on testis of lead-exposed mice. Moreover, the supplementation of aqueous coriander extract would also provoke an increase in sperm density, compared with lead nitrate-treated group.

The biological effects of the WBV exercises in an organ seem to be also dependent on the frequency [16,22]. Miyazaki, 2000 has evaluated the electrogastrography (EGG) in healthy male volunteers’ exposure to vibrations of 4, 8, and 16 Hz. This author has observed that only the vibrations of 4 and 8 Hz have decreased the amplitude of the EGG.

The determination of the effect of 40-day WBV (50 Hz) on the level of plasma biomarkers in rats treated with coriander (Table 2) has shown no alterations on the concentrations of the studied biomarkers. Although the protocols with WBV used by other authors were not exactly the same that was used in the present study, it is shown an agreement with Pawlak et al. [18] that showed low-volume WBV (50 Hz) lasting 3 or 6 months does not affect biomarkers in blood serum of rats, using the same frequency. Moreover, the levels of the studied biomarkers were also consistent. Naghii et al. [17], using a different protocol, have determined the plasma lipid concentrations (total cholesterol, low-density lipoprotein (LDL), and HDL) in rats submitted to vibrations in the frequencies of 10–50 Hz and they did not found alteration in the concentrations of these biomarkers, although they have found significant differences in plasma levels of CK. The plasma CK levels were significantly higher in the vibration group compared with the controls.

The comparison of the animals among the groups has shown that there is no significant alteration on the body mass (Table 3) in rats treated 40 days simultaneously with coriander and WBV exercise. It is important to point out that, this finding is not associated with the feed intake, which was increased in rats exposed to the WBV exercise. It is very interesting and could associated with the results reported by Wang and Kerrick et al. [44] that verified that applying vibration to intact or skinned single fiber preparations occurs specific increase in ATP turnover. These results are in agreement with Huang et al. [45] that have reported in mice with obesity induced by a high-fat diet (HFD), no difference in body mass between HFD with sedentary control, HFD with WBV at relatively low-intensity (5.6 Hz, 0.13 g) (HFD + VL) or high-intensity (13 Hz, 0.68 g) (HFD + VH). In addition, Di Loreto et al. [46] reported that since hormonal responses, with the exception of norepinephrine, are not affected by acute WBV exposure, this type of exercise is not expected to reduce fat mass.

The effect of the association of 40-day WBV (50Hz) with coriander (Figure 4) on the feed intake in rats has shown alterations among the groups. An increase on the feed intake was found in animals of the WBV-E (Figure 4). This result differs from Frederico et al. [17] that reported no difference in the amount of feed between the groups. Huang et al. [45] also shown no difference on energy intake among the groups studied using a different protocol.

The stool consistency of animals submitted to different treatment (Table 4) permits to verify that rats that consumed coriander alone (COR) has the stool consistency classified as type 1. In the animals that are submitted to WBV associated with coriander (COR + WBV-E), the stool consistency is normalized (type 2). This finding indicated that the WBV would act in some physiological/metabolic step in the gastrointestinal system that would be related to the normalizing of the stool consistency. Considering the gastrointestinal system, Ishitake et al. [47] have reported that WBV suppresses gastric motility in healthy men. In addition, the hard stool (type 1) in animals treated with coriander can be justified due to the effect of this natural product that is used in traditional medicine for the treatment of gastrointestinal diseases, such as diarrhea [37].

The present study has some limitations, as the small number of animals in each group. However, it was followed the criteria of reduction strategies in animal research, published by [48]. Moreover, a special type of cage was not used to separate the stools and it was difficult to monitor the weight of the stools. The WBV-treated rats were immobilized at the platform. This could induce immobility stress, which potentially may alter some physiological parameters of the rats. As the control rats were not submitted to immobility stress, the plasma concentration of specific biomarkers related to stress could be considered in the present study. In addition, the suggestion of new experimental models involving the exposition of animals to the (i) WBV exercise generated by mechanical vibrations with different biomechanical parameters (frequency, peak-to-peak displacement, and peak acceleration) and several times of exposition and (ii) various synthetic and natural products are desirable to verify possible metabolic responses.

Despite the limitations and putting together all the findings, it is concluded that possible modifications in some biochemical/physiological parameters of the rats submitted to WBV exercise would be capable to increase the feed intake without changing the body mass, and normalizing the stool consistency altered by the coriander supplementation. Further studies are needed to try to understand better the biological effects of involving the association of WBV exercise and an aqueous coriander extract.

## Abbreviations

%ATI/g: percentages of injected radioactivity per gram
ALT: alanine aminotransferase
AST: aspartate aminotransferase
CK: creatine kinase
CNPq: *Conselho Nacional de Pesquisa e Desenvolvimento*
CON: control group
CONCEA: *Conselho Nacional de Controle de Experimentação Animal*
COR: group treated with coriander
COR + WBV-E: group treated with coriander and submitted to the vibration generated in the platform
EGG: electrogastrography
*ɛ*^2^: epsilon-squared
FAPERJ: *Fundação de Amparo à pesquisa do Estado do Rio de Janeiro*
HDL: high-density lipoprotein
HFD: high-fat diet
HFD + VH: high-fat diet with whole body vibration at high-intensity
HFD + VL: high-fat diet with whole body vibration at relatively low-intensity
IQR: interquartile range
LDL: low-density lipoprotein
*P*: *P-*value
SD: standard deviation
UERJ: *Universidade do Estado do Rio de Janeiro*
WBV: whole body vibration
WBV-E: group of rats submitted to the vibration generated in the platform
